# Leucine-Enriched Essential Amino Acids in Clinical Practice: A Narrative Review

**DOI:** 10.3390/nu18183024

**Published:** 2026-09-16

**Authors:** Kenji Nagao, Tatsuya Hasegawa, Hideaki Kihara, Natsumi Nishikata, Hisamine Kobayashi, Philip J. Atherton, Satoshi Fujita

**Affiliations:** 1 Amino Acids Department, Bio & Fine Chemicals Division, Ajinomoto Co., Inc., 7-1 Kyobashi 1-Chome, Chuo-ku, Tokyo 104-0031, Japan; tatsuya.hasegawa.vp4@asv.ajinomoto.com; 2 Research & Business Department, Corporate Division, Ajinomoto Co., Inc., 7-1 Kyobashi 1-Chome, Chuo-ku, Tokyo 104-0031, Japan; hideaki.kihara01.ib5@asv.ajinomoto.com or hideaki.kihara@vitalnutri.com (H.K.); natsumi.nishikata.rr6@asv.ajinomoto.com (N.N.); 3 Business Strategy & Planning Department, Bio & Fine Chemicals Division, Ajinomoto Co., Inc., 7-1 Kyobashi 1-Chome, Chuo-ku, Tokyo 104-0031, Japan; hisamine.kobayashi.f2x@asv.ajinomoto.com; 4 Department of Sports and Health Sciences, Graduate School of Biomedical Engineering, Tohoku University, 2-1 Seiryo-machi, Aoba-ku, Sendai 980-8575, Japan; 5 Centre of Metabolism, Ageing and Physiology (CoMAP), Academic Unit of Injury, Recovery and Inflammation Sciences (IRIS), School of Medicine, University of Nottingham, Royal Derby Hospital, Uttoxeter Road, Derby DE22 3DT, UK; philip.atherton@nottingham.ac.uk; 6 Faculty of Sport and Health Science, Ritsumeikan University, 1-1-1 Noji-higashi, Kusatsu 525-8577, Japan; safujita@fc.ritsumei.ac.jp

**Keywords:** leucine, essential amino acids, sarcopenia, hemodialysis, cachexia, clinical nutrition, mTORC1, protein turnover, muscle protein synthesis

## Abstract

Muscle wasting driven by aging, chronic disease, or intensive exercise undermines mobility, metabolic health, and survival—yet the people who lose the most muscle are often those least able to eat more protein. Leucine-enriched essential amino acid (LEAA) mixtures combine a leucine signal that switches on muscle protein synthesis with the indispensable amino acids needed to sustain it. This narrative review examines 50 human studies. LEAA mixtures are defined here as oral supplements containing all nine indispensable amino acids with about 40% or more leucine by weight; studies of leucine-enriched branched-chain amino acids, free leucine, and leucine-enriched protein are treated as indirect evidence. The clearest findings are in older adults: 1.5–3 g raised muscle protein synthesis as much as 20–40 g of whey protein, an effect out of proportion to the amount given, and it was the leucine proportion, not the total amino acid content, that determined the response. Adding free leucine to a diet already sufficient in protein gave no further benefit, so the efficiency appears to lie in the combination rather than in leucine alone. Function improved when LEAA was combined with exercise, but exercise was given in all such trials, so the effect of LEAA alone is unclear. Evidence for long-term benefits that patients can feel is still limited, and safety has not been assessed systematically. Delivering the same stimulus in a few grams is therefore the main clinical argument for LEAA, and it now needs testing.

## 1. Introduction

Leucine, a branched-chain amino acid (BCAA), is a potent driver of muscle protein synthesis via activation of the mammalian target of rapamycin complex-1 (mTORC1) signaling pathway [1]. In aging and chronic disease, muscle responds less to food than it should—a state known as anabolic resistance—and muscle is gradually lost, with function declining alongside it. This matters because the populations most affected are frequently those least able to increase protein intake. Reduced appetite, early satiety, altered taste, dysphagia, and fluid or protein restrictions are common in older adults and in patients with cancer, liver disease, or chronic kidney disease, and a nutritional strategy that delivers an anabolic stimulus in a few grams rather than tens of grams would therefore address a practical as well as a physiological constraint.

LEAA mixtures were developed to counteract anabolic resistance: leucine activates anabolic signaling, and the essential amino acids supply the substrate that net protein synthesis needs [2,3]. That the mixture matters, and not leucine alone, is supported by two observations examined in this review: when total amino acid content is held constant, leucine proportion determines the anabolic response [4], whereas adding free leucine to an already sufficient protein intake does not [5].

In this review, we define “leucine-enriched essential amino acid (LEAA) mixtures” as oral supplements that supply all nine indispensable amino acids and in which leucine constitutes approximately 40% or more of total amino acid content by weight. We adopt this threshold as an operational convention for the present review, not as an established biological or clinical cut-off. It is anchored to a single controlled metabolic study in which an EAA mixture enriched to approximately 41% leucine increased muscle protein synthesis in older adults whereas an isonitrogenous mixture containing approximately 26% leucine did not, with no comparable difference in younger participants [4]. That study compared two discrete leucine proportions in 36 participants and did not identify a threshold; no dose–response study has established the leucine proportion at which the anabolic response changes, and no evidence indicates that a single proportion would apply across populations, disease states, or total amino acid doses. The value of approximately 40% is used here because it corresponds to the composition of the formulations most commonly studied and therefore permits consistent classification of the literature.

This operational definition distinguishes LEAAs from four related but distinct interventions, which are classified separately throughout this review (Section 2.4): (i) leucine-enriched branched-chain amino acid (BCAA) preparations, which supply only leucine, isoleucine, and valine; (ii) leucine-enriched protein formulations, in which free leucine is added to an intact protein source such as whey; (iii) free leucine given as a single amino acid; and (iv) formulations providing a selected subset of the essential amino acids rather than all nine. Some studies employed formulations with leucine fractions just below 40% (e.g., 35% [6]). We discuss these studies as near-LEAA evidence when they are explicitly described as leucine-enriched EAA mixtures, while noting their composition. The classification is also sensitive to how the leucine fraction is expressed: in one trial the preparation provided leucine at 37.9% of total amino acid content but 41.6% of the nine indispensable amino acids, and is classified here as near-LEAA on the former basis [7].

**Figure 1:**
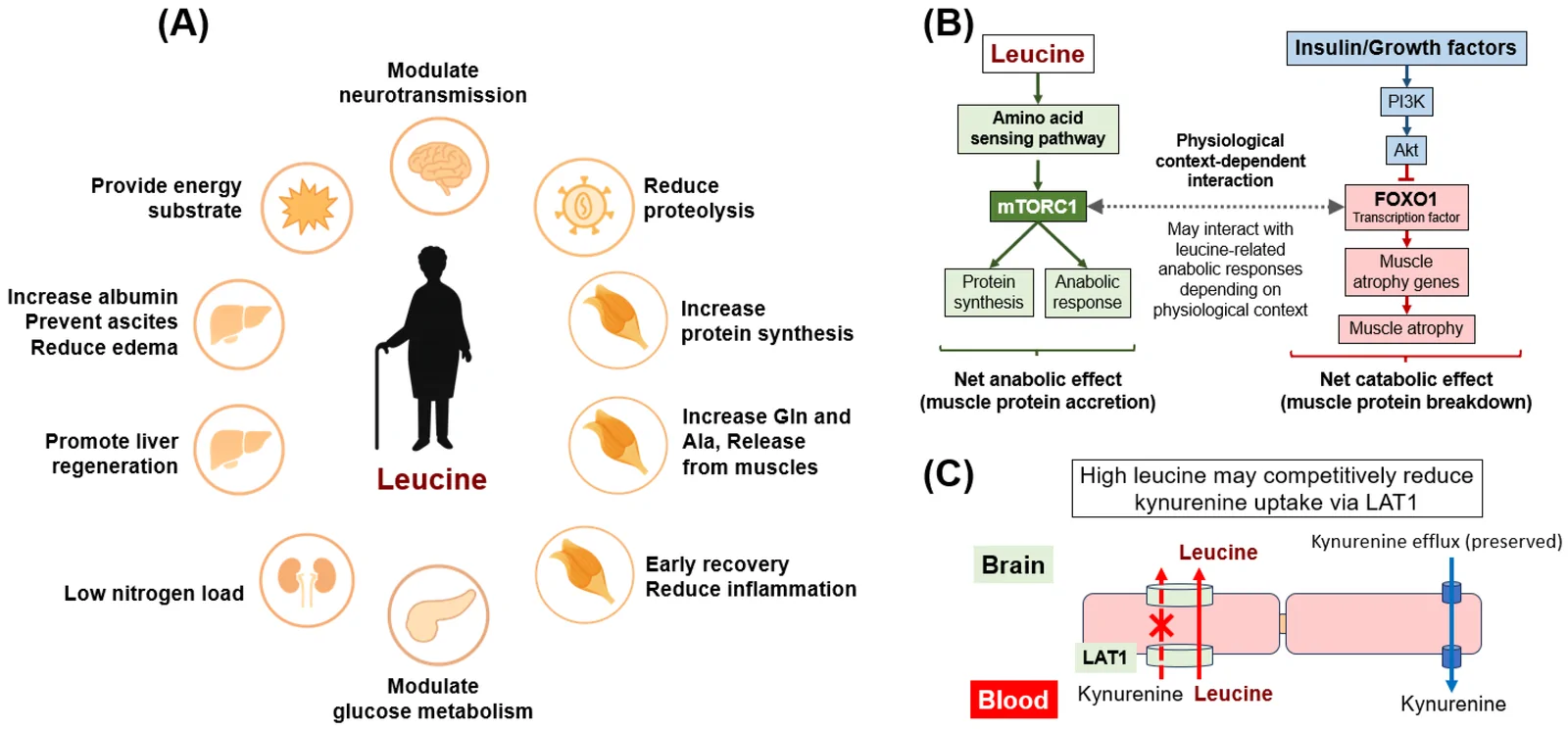
The mechanistic and clinical effects associated with leucine, BCAA, and LEAA interventions. (**A**) The physiological and clinical effects attributed to leucine, leucine-enriched BCAA, and LEAA interventions across organs and tissues. The effects shown derive from different intervention types and are not all attributable to leucine itself; in particular, the hepatic effects (increased serum albumin, prevention of ascites, reduction in edema, promotion of liver regeneration, and low nitrogen load) derive from studies of leucine-enriched BCAA in cirrhosis (Section 4.3.1) and have not been demonstrated for LEAA mixtures. (**B**) Leucine is proposed to regulate mTORC1 through an amino acid sensing pathway, whereas the Akt–FOXO1 axis is predominantly regulated by insulin and growth factor signaling. Akt activation suppresses FOXO1, thereby restraining the catabolic cascade; conversely, when Akt signaling is low, FOXO1 is de-repressed and induces muscle atrophy. This axis may interact with leucine-related anabolic responses in a physiological context-dependent manner, as indicated within the panel. (**C**) High plasma leucine may competitively reduce LAT1-mediated kynurenine uptake at the blood–brain barrier (dashed arrow) whereas kynurenine efflux (solid blue arrow), which is largely independent of LAT1 and not inhibited by leucine, is preserved; this may lower neuroinflammatory kynurenine in the brain [8]. The cross denotes competitive reduction in kynurenine influx rather than complete blockade. This mechanism derives from preclinical (murine and in vitro) data and is proposed rather than established in humans. LEAA, leucine-enriched essential amino acid; BCAA, branched-chain amino acid; PI3K, phosphoinositide 3-kinase; Akt, protein kinase B; mTORC1, mammalian target of rapamycin complex 1; FOXO1, forkhead box protein O1; LAT1, L-type amino acid transporter 1; Gln, glutamine; Ala, alanine. The evidence summarized in this figure is derived from heterogeneous sources, including LEAA mixtures, leucine-enriched BCAA, free leucine, and preclinical studies; not all effects have been demonstrated specifically for LEAA mixtures in clinical trials.

The clinical literature on leucine-enriched amino acid interventions is compositionally heterogeneous. Over the past two decades, clinical and mechanistic studies have examined LEAA mixtures and related interventions in diverse populations, including older adults with sarcopenia, individuals undergoing exercise or rehabilitation, and patients with liver cirrhosis, chronic kidney disease (CKD), cancer, neurological conditions, and other emerging indications (Figure 1A). In several therapeutic areas, most notably liver cirrhosis, clinically important evidence has been generated predominantly with leucine-enriched BCAA rather than full-spectrum LEAA mixtures; in others, it comes from free leucine or leucine-enriched protein formulations. We therefore include these related interventions only when they are mechanistically relevant or clinically informative, and we distinguish them clearly from direct LEAA evidence, treating them as supportive or hypothesis-generating rather than interchangeable. The strength and directness of the evidence vary substantially by formulation, population, co-intervention, and endpoint (Table 1 and Table 2).

Several prior reviews have addressed branched-chain amino acid and leucine supplementation in chronic liver disease [53], the effects of leucine supplementation on the criteria that define sarcopenia [44], and the comparative efficacy of nutritional interventions for sarcopenia in cirrhosis [45]. However, the clinical evidence for LEAA mixtures, defined by an explicit compositional threshold and distinguished from adjacent leucine-enriched interventions, has not been synthesized across these clinical contexts. Therefore, this narrative review aims to map the evidence landscape for LEAA mixtures in clinical practice, to identify where formulation-specific evidence is relatively robust, and to clarify where conclusions rely on extrapolation from related interventions. On that basis, it further aims to identify the populations in which the compact format of these preparations is most likely to matter clinically, and the trial design that would test it.

Throughout the review, we distinguish direct LEAA evidence from indirect or supportive evidence and interpret findings in light of study design, intervention type, co-interventions, and endpoint hierarchy where available.

## 2. Methods

### 2.1. Review Design and Scope

This article was designed as a narrative review rather than a systematic review or meta-analysis. The review covers 50 human studies, of which 19 used formulations meeting the LEAA definition; their distribution across clinical indications is shown in Table 2. The primary focus was clinical and mechanistic evidence for LEAA mixtures, as defined in Section 1. Related leucine-enriched interventions were considered where mechanistically relevant or clinically informative, and are classified separately (Section 2.4).

### 2.2. Literature Search

PubMed (MEDLINE) and Google Scholar were searched on 3 September 2026, from database inception. Search strategies were constructed around a common intervention block (leucine, essential amino acids, leucine-enriched essential amino acid, LEAA, branched-chain amino acid, BCAA) combined in turn with blocks representing the eight mechanistic and clinical domains covered by this review, which are listed with the records each returned in Supplementary Methods S1. The eight searches returned 60,122 records, or 54,063 after removal of overlapping studies. Species and language restrictions were applied at the screening stage rather than as search filters, for the reasons given in Supplementary Methods S1. The complete database-specific search strings, the date on which each was executed, and the number of records retrieved are provided in Supplementary Methods S1. Because Google Scholar does not support reproducible Boolean syntax or stable result counts, it was used only as a supplementary source. Additional studies were identified from the reference lists of key publications and from the authors’ prior knowledge of the field. Supplementary Methods S1 reports how many of the tabulated studies were identified by each route: 47 of 50 were retrieved by the database searches. Priority was given to human intervention studies published in English. Preclinical studies were not searched systematically and were cited only where required to support mechanistic interpretation in Section 3.

### 2.3. Eligibility Considerations

This review did not employ a protocol-registered screening process, and study selection was not performed in duplicate or formally documented at the time of the search. The following considerations were applied when deciding whether a retrieved record was discussed in this review, and are stated here to make the basis of inclusion explicit rather than to imply a systematic selection procedure.

Studies were considered eligible for discussion if they (i) administered an LEAA mixture or one of the related preparations defined in Section 1, orally or enterally; (ii) enrolled human participants; (iii) reported outcomes relating to muscle protein synthesis or turnover, body composition, muscle strength, physical function, quality of life, neurological outcomes, or disease-related clinical endpoints; and (iv) were published in English as full papers in peer-reviewed journals.

Records were not discussed if they (i) evaluated intravenous or parenteral amino acid administration only; (ii) reported no amino acid intervention (observational or purely dietary-pattern studies); (iii) were conference abstracts, case reports, or narrative commentaries without primary data; or (iv) were animal or in vitro studies, except where cited in Section 3 to support a mechanistic point.

Studies reporting negative or null findings were eligible on the same basis as those reporting positive findings, and randomized trials in which the prespecified primary endpoint was not met are discussed explicitly in Section 4 and tabulated in Supplementary Table S1 [5,13,16,21,24,26,28,38,39,54,55].

### 2.4. Intervention-Based Classification

To address heterogeneity in the literature, studies were interpreted according to intervention type. We distinguished six intervention-based categories: (i) direct LEAA evidence, defined as studies using mixtures containing all nine indispensable amino acids with leucine accounting for approximately 40% or more of total amino acid content; (ii) near-LEAA evidence, defined as mixtures containing all nine essential amino acids with leucine enrichment modestly below this threshold (approximately 34–38%); (iii) leucine-enriched BCAA evidence; (iv) free leucine evidence; (v) leucine-enriched protein evidence; and (vi) selected EAA formulation evidence, defined as mixtures containing only a selected subset of essential amino acids rather than all nine (e.g., a seven-essential-amino-acid formulation). One additional descriptive label, “mixed EAA plus intact protein,” was applied to a formulation that combined essential amino acids with intact protein and whose leucine fraction was not reported. Because Supplementary Table S1 catalogs human studies only, mechanistic and preclinical evidence was not treated as a separate tabulated category; instead, it is discussed primarily in Section 3, and human studies whose primary endpoints were intracellular signaling rather than clinical or functional outcomes are flagged with a “(mechanistic)” descriptor. This classification was used to avoid overgeneralizing findings from related interventions to LEAA mixtures.

Throughout Section 4, each study is annotated at first mention with its intervention category. Section 4 is organized at the first level according to whether direct LEAA evidence is available for a given indication (Section 4.2) or whether the available evidence derives entirely from related leucine-enriched interventions (Section 4.3); the distribution of intervention categories across indications is shown in Table 2. Conclusions specific to LEAA mixtures are drawn in Section 4.4, which is restricted to studies meeting the operational LEAA definition.

### 2.5. Data Interpretation and Synthesis

For each study discussed, we considered the population, intervention type, leucine dose or proportion where available, comparator, duration, co-interventions, and reported outcomes. When available, primary and secondary endpoints were distinguished. Findings based only on secondary endpoints, within-group changes, exploratory analyses, or studies without significant between-group differences were interpreted cautiously. Because of substantial heterogeneity in study populations, formulations, doses, timing, co-interventions, and outcomes, no quantitative meta-analysis was performed. Instead, findings were synthesized narratively, with emphasis on the strength, directness, and clinical relevance of the evidence for each indication.

To make the basis of these judgments explicit, two independent descriptors were applied to every study in Supplementary Table S1. A design tier reflects internal validity for comparative inference, from confirmatory (randomized and controlled, at least 100 participants, with a prespecified primary endpoint) to preliminary (non-randomized or uncontrolled); the four levels are defined in Supplementary Table S1. An endpoint level reflects clinical proximity, from mechanistic signaling through surrogate physiological measures to intermediate clinical and patient-important outcomes; the four levels are likewise defined in Supplementary Table S1. The two descriptors are deliberately independent, because a single composite rating would conflate internal validity with clinical relevance: acute stable-isotope tracer studies are necessarily small and therefore fall into tier 3 or 4, which reflects their limited capacity to support comparative clinical inference rather than their suitability for mechanistic inference. Supplementary Table S1 additionally records, for each study, the prespecified primary endpoint and whether it was met, the principal limitations, and the safety and tolerability information reported. These descriptors are intended as a transparent indication of evidence adequacy and do not constitute a formal risk-of-bias or certainty-of-evidence assessment.

## 3. Mechanisms of Action

The biological rationale for LEAA supplementation rests on two complementary mechanisms: leucine-mediated activation of anabolic signaling and provision of all indispensable amino acid substrates required for net protein synthesis. Leucine is not only an essential amino acid and a component of BCAAs, but also a nutrient signal that regulates skeletal muscle protein metabolism. It activates mTORC1 signaling through dedicated amino acid-sensing pathways, including interactions with Sestrin 1 and Sestrin 2, and is associated with phosphorylation of downstream targets such as S6K1 and 4E-BP1 [1]. Through these pathways, leucine can promote translation initiation and muscle protein synthesis. The Akt–FOXO1 axis, which restrains FOXO1-mediated catabolic signaling and autophagy-related pathways, is predominantly regulated by insulin and growth-factor signaling rather than by leucine directly; leucine-related anabolic responses may interact with this axis in a physiological context-dependent manner (Figure 1B), although the relative contribution of each pathway may differ by physiological and disease context.

Importantly, leucine signaling alone is insufficient to sustain net protein accretion unless other indispensable amino acid substrates are available. This distinction is central to the rationale for LEAA mixtures. Whereas BCAA formulations provide only leucine, isoleucine, and valine, LEAA mixtures are designed to combine a strong leucine signal with the full set of indispensable amino acid substrates required for complete protein synthesis. In resistance-exercise studies, leucine-enriched EAA intake increased muscle protein synthesis and mTOR-related signaling [56], while leucine was required for maximal S6K1 activity and acted more effectively in the presence of other EAAs [50]. Thus, the mechanistic advantage of LEAA mixtures should be understood as the combination of leucine-mediated signal activation and essential amino acid substrate sufficiency, rather than as an effect of leucine alone (Figure 1B). This division of labor also explains why the effective amounts are small. Leucine acts as a signal rather than as bulk substrate, and a signal is saturable: once the sensing pathway is engaged, additional leucine adds little. The substrate requirement is set by the amount of protein actually synthesized over the following hours, which is modest. A preparation that supplies a saturating leucine signal together with the corresponding indispensable amino acid substrate can therefore be far smaller than an intact protein bolus delivering the same anabolic effect, most of which is neither a signal nor an incorporated substrate. Consistent with this interpretation, a double-blind trial in pre-frail and frail older women whose protein intake was optimized to 1.2 g/kg/day found that adding 7.5 g/day of free leucine to a twelve-week resistance training program produced no additional increase in myofibrillar protein synthesis over an isonitrogenous alanine comparator, indicating that leucine confers little further anabolic benefit when indispensable amino acid substrate is already sufficient (Section 4.2.1) [5].

Human tracer studies support this concept, particularly in older adults with anabolic resistance. In older individuals, EAA mixtures containing a high proportion of leucine induced greater muscle protein synthesis than isonitrogenous mixtures with lower leucine content, whereas no comparable difference between leucine proportions was observed in younger individuals [4]. These findings suggest that leucine enrichment may be most relevant in states where the anabolic response to ordinary protein or amino acid intake is blunted, such as aging, chronic disease, inactivity, or rehabilitation. Similarly, in active young adults, an EAA mixture enriched to 35% leucine increased post-exercise muscle protein synthesis and suppressed whole-body protein turnover compared with an isonitrogenous EAA mixture containing a lower leucine proportion, although mTORC1 pathway phosphorylation did not differ between treatments [6]. Because this formulation contained 35% leucine, it is best interpreted as near-LEAA mechanistic evidence rather than direct evidence under the operational definition used in this review.

Evidence from catabolic disease states is supportive but should be interpreted carefully according to formulation type. In men with advanced non-small-cell lung cancer, a 14 g essential amino acid mixture enriched to 40% leucine increased whole-body protein synthesis and improved net protein anabolism compared with a balanced amino acid mixture containing both essential and non-essential amino acids [43]. However, the authors attributed the superior anabolic response primarily to the higher total EAA content rather than to leucine enrichment per se. This finding supports the importance of EAA provision in catabolic disease, but it does not isolate the independent contribution of leucine enrichment above a standard EAA formulation.

Related evidence supports biological plausibility without being equivalent to direct LEAA evidence. In alcoholic cirrhosis, a single oral dose of leucine-enriched BCAA restored defective mTORC1 signaling and reduced autophagy markers in skeletal muscle [51]; the preparation did not supply all nine indispensable amino acids, so this speaks to leucine-sensitive pathways rather than to LEAA mixtures. Leucine also has a modest insulinotropic effect, and intragastric leucine raised circulating insulin in healthy lean subjects [52], which may aid amino acid uptake under some conditions; its clinical relevance is likely context-dependent and should not be assumed to explain the effects described here.

Beyond skeletal muscle, emerging mechanistic work has suggested possible effects of leucine and selected essential amino acids on neurological pathways. One proposed mechanism is competition between circulating large neutral amino acids, including leucine, and kynurenine for LAT1-mediated transport across the blood–brain barrier. By limiting brain kynurenine influx, leucine or EAA supplementation may theoretically reduce neuroinflammatory signaling and support neurotransmitter balance (Figure 1C) [8,57]. Preclinical studies also suggest that EAA supplementation can influence neurotransmitter pools and neurological phenotypes [58,59]. However, these mechanisms remain largely preclinical or hypothesis-generating and should be distinguished from established clinical efficacy evidence.

Taken together, LEAA mixtures are mechanistically attractive because they combine leucine-driven anabolic signaling with complete essential amino acid substrate availability. The strongest direct mechanistic evidence relates to stimulation of muscle protein synthesis in older adults and in response to exercise. Evidence from near-LEAA formulations, BCAA, free leucine, leucine-enriched protein, and preclinical neurological studies provides useful biological context, but these findings should not be overgeneralized to LEAA mixtures without formulation-specific clinical confirmation. This dual requirement—a leucine signal sufficient to activate translation initiation, together with the full complement of indispensable amino acids to sustain it—is the specific hypothesis that the clinical literature examined in the following section is used to test. The sections that follow are organized so that evidence bearing directly on that hypothesis can be distinguished from evidence bearing on it only indirectly.

## 4. Clinical Evidence: Indication-Based Synthesis Stratified by Evidence Directness

### 4.1. Overview of Evidence Landscape

Before considering individual indications, it is useful to summarize the compositional basis of the available literature, because the distribution of intervention types across clinical fields is markedly uneven. Table 2 maps the 50 human studies cataloged in Supplementary Table S1 onto the intervention categories defined in Section 2.4.

The direct LEAA evidence is not evenly spread but is concentrated: nine of the nineteen studies meeting the definition were conducted in older adults, either as acute tracer studies of muscle protein synthesis or as trials combining supplementation with exercise, and it is in this population that the findings are most consistent. Two features of this distribution shape the interpretation of the sections that follow. First, studies using formulations that meet the operational LEAA definition account for 19 of the 50 tabulated studies, and these are unevenly distributed: seven indications are supported by at least one direct LEAA study, but in three of them the direct evidence consists of a single trial. Second, two indications discussed in this review—liver cirrhosis and heart failure—contain no direct LEAA evidence at all. In liver cirrhosis, which is the most extensively studied field in this literature and the only one addressed by clinical practice guidelines, all nine tabulated studies used leucine-enriched BCAA rather than a full-spectrum LEAA mixture.

Section 4 is therefore organized at the first level according to whether direct LEAA evidence exists for a given indication. Section 4.2 covers indications with at least one direct LEAA study, and Section 4.3 covers indications in which the available evidence derives entirely from related leucine-enriched interventions. Each subsection opens with a statement of its evidence base, and each study is annotated at first mention with its intervention category. Section 4.4 then synthesizes the direct LEAA evidence alone; the conclusions of this review (Section 7) are derived from that section.

### 4.2. Indications with Direct LEAA Evidence

The indications in this section are supported by at least one study using a formulation meeting the operational LEAA definition (approximately 40% or more leucine, all nine indispensable amino acids). The amount and quality of that direct evidence nevertheless vary widely, from four acute tracer studies in older adults to single small trials in hemodialysis, orthopedic rehabilitation, and cognitive impairment. Evidence from related interventions is discussed within each subsection where mechanistically or clinically informative, but is identified as such and is not used to support LEAA-specific conclusions.

#### 4.2.1. Muscle Protein Synthesis and Body Composition in Older Adults

Evidence base: direct LEAA, 4 studies; near-LEAA, 1; free leucine, 2; leucine-enriched protein, 1 (Table 2). This is the indication with the largest body of direct LEAA evidence, although three of the four direct studies are acute tracer studies and the longest direct LEAA exposure reported is 12 weeks.

Human tracer studies provide some of the most direct evidence that LEAA mixtures can efficiently stimulate muscle protein synthesis in older adults. Aging muscle is characterized by anabolic resistance, in which the response to ordinary protein or amino acid intake is blunted. In this context, leucine enrichment appears particularly relevant. In older adults, an EAA mixture containing 41% leucine significantly increased muscle protein synthesis, whereas an isonitrogenous mixture containing 26% leucine showed limited effectiveness [4]. In contrast, younger individuals showed comparable responses regardless of leucine proportion, suggesting that the benefit of leucine enrichment is most apparent in aging muscle with impaired anabolic sensitivity [4].

This concept is further supported by studies showing that increased leucine availability can enhance postprandial myofibrillar protein synthesis in older adults. Supplementation of a mixed meal with free leucine significantly increased postprandial myofibrillar protein fractional synthesis rate in healthy older men without altering whole-body protein kinetics [60]. Although this study used free leucine rather than an LEAA mixture, it supports the biological premise that leucine availability is a key determinant of anabolic responsiveness in older muscle. The LEAA strategy builds on this premise by combining a leucine signal with all indispensable amino acid substrates required for net protein synthesis.

Low-dose LEAA studies are particularly important because they suggest that high-leucine EAA formulations can stimulate muscle protein synthesis with substantially smaller amino acid loads than intact protein. In older women, 3 g of LEAA containing 1.2 g leucine stimulated muscle anabolism to a degree comparable to 20 g of whey protein, both at rest and after exercise [9]. A subsequent dose–response study reported that as little as 1.5 g of LEAA containing 0.6 g leucine stimulated muscle protein synthesis at rest and after resistance exercise, with responses comparable to those observed after larger whey protein boluses [10]. These findings support the practical advantage of LEAA mixtures as compact, low-volume nutritional interventions for older adults who may have reduced appetite, early satiety, or difficulty consuming larger protein loads.

Together, the acute tracer studies indicate that both leucine proportion and total indispensable amino acid composition matter in aging muscle. Formulations containing approximately 40% leucine appear able to trigger anabolic responses at low total amino acid doses, whereas lower-leucine isonitrogenous mixtures may be less effective in older adults [4,9,10]. At the same time, these studies should not be interpreted as showing that leucine alone is sufficient. Rather, they support the concept that efficient stimulation of muscle protein synthesis requires both leucine-mediated signaling and adequate provision of essential amino acid substrates.

The specificity of leucine as the active component has been directly questioned. In a double-blind trial in pre-frail and frail older women whose protein intake was optimized to 1.2 g/kg/day, twelve weeks of resistance training increased myofibrillar fractional synthesis rate by 47% and reduced the number of frailty criteria by 64%, but the addition of 7.5 g/day of free leucine conferred no further anabolic benefit over an isonitrogenous alanine comparator [5]. This finding is consistent with the interpretation that the contribution of LEAA mixtures lies in the provision of the full indispensable amino acid substrate rather than in leucine alone.

Longer-term studies suggest that these acute anabolic effects may translate into improvements in body composition and selected functional outcomes, although the evidence is more heterogeneous. In a 12-week double-blind placebo-controlled pilot trial in adults aged 65–75 years, twice-daily supplementation with an EAA mixture containing 40% leucine increased lean tissue mass from baseline, and both the 40% and the 20% leucine mixtures improved functional performance from baseline; however, neither prespecified primary outcome differed between groups, and no additional effect was observed with the higher leucine proportion [11]. The authors attributed the absence of a between-group effect to the adequacy of habitual protein intake in their participants, which averaged 1.0–1.1 g/kg/day, noting that when protein and leucine intakes are already optimal additional supplementation may confer no further benefit [11]—the same interpretation later supported experimentally by the free-leucine trial described above [5]. They also reported 30% attrition, attributed in part to difficulty taking the supplement at doses of 11–21 g/day, and concluded that formulations delivering essential amino acids in a more compact and palatable form were needed. In glucose-intolerant older adults, twice-daily near-LEAA mixture (~36% leucine) plus arginine for 16 weeks was associated with improvements in lean body mass, strength, and physical function from baseline [14]. In addition, an uncontrolled pilot study using leucine-enriched whey protein plus vitamin D during caloric restriction in sarcopenic obese women preserved lean mass and improved handgrip strength and short physical performance battery (SPPB) scores [61]. This latter formulation falls outside the LEAA definition used in this review, but it provides supportive evidence that leucine-enriched nutritional strategies may be useful during catabolic or energy-restricted states.

Overall, aging muscle is one of the areas in which the rationale and evidence for LEAA mixtures are most coherent. Direct tracer studies consistently support efficient stimulation of muscle protein synthesis by low-dose, leucine-enriched EAA formulations, and longer-term clinical studies suggest potential benefits for lean mass and physical performance. However, larger trials with predefined functional endpoints are still needed to determine how these acute anabolic effects translate into durable improvements in strength, mobility, and clinically meaningful outcomes.

#### 4.2.2. LEAA Combined with Exercise or Rehabilitation in Older Adults

Evidence base: direct LEAA, 5 studies; near-LEAA, 1; leucine-enriched BCAA, 1; leucine-enriched protein, 1 (Table 2). All direct LEAA studies administered supplementation together with structured exercise or rehabilitation, and none included an LEAA-only arm, so the independent contribution of LEAA cannot be separated from that of the co-intervention. That every trial combined the two reflects the underlying rationale rather than an oversight: resistance exercise provides the anabolic stimulus, and the amino acid mixture provides the substrate that stimulus requires, and neither is expected to be sufficient alone in a population with anabolic resistance. The findings below should therefore be read as evidence for multimodal programs containing LEAA rather than for LEAA itself. That every trial in this indication combined supplementation with exercise reflects the underlying rationale rather than an oversight. Resistance exercise provides the anabolic stimulus, and the amino acid mixture provides the substrate that stimulus requires; neither is expected to be sufficient alone in a population with anabolic resistance.

Among community-dwelling older Japanese women with sarcopenia, a 3-month intervention combining exercise with twice-daily intake of 3 g LEAA (1.26 g leucine) significantly improved muscle strength and composite outcomes incorporating muscle mass, strength, and walking speed compared with other groups [15]. In another randomized trial of older Japanese women with low muscle mass but preserved strength, adding 3 g LEAA (1.2 g leucine) to a once-weekly exercise program did not significantly enhance primary endpoints (muscle mass or strength), but did alleviate low-back discomfort as a secondary endpoint [13]. Similarly, supplementation with 3 g or 6 g LEAA containing approximately 40% leucine, combined with a training program targeting psoas major hypertrophy, improved walking ability in older Japanese adults [12]. Because exercise was delivered in every trial, these findings do not permit a comparison between LEAA with and without mechanical loading, and the magnitude of benefit likely depends on baseline frailty, exercise intensity, and outcome selection.

Clinical rehabilitation studies provide further supportive evidence. In post-stroke patients with sarcopenia, an 8-week intervention combining 3 g LEAA (1.2 g leucine) with low-intensity resistance training met its prespecified primary endpoint (motor-domain Functional Independence Measure) versus control, with secondary gains in grip strength and skeletal muscle mass index; the trial was open-label with blinded outcome assessment [30]. Another study using a leucine-enriched BCAA formulation providing 12 g/day (6 g BCAA with ~3 g leucine, twice daily; ~6 g leucine/day) in combination with comprehensive rehabilitation reported beneficial effects on stroke-related sarcopenia and neurological recovery [31]. Because this latter intervention did not provide all nine indispensable amino acids, it should be interpreted as related BCAA evidence rather than direct LEAA evidence. This dual-intervention model, in which exercise provides the primary anabolic stimulus while LEAA supplementation supplies amino acid availability during recovery, is mechanistically plausible but has not been tested against exercise alone in a design capable of isolating the amino acid contribution.

A subsequent open-label randomized trial in 66 healthy older adults compared LEAA plus resistance training with LEAA plus walking and general strengthening over six months. Both groups received the supplement, and the prespecified primary outcome of appendicular skeletal muscle mass index did not improve in either group; gait speed improved in both, without a between-group difference. Adverse events were collected prospectively and renal function was monitored, with no serious events reported [16]. A four-arm trial in 96 older women found that resistance exercise combined with a near-LEAA mixture improved muscle mass and functional test scores more than either component alone [7]; the preparation contained arginine and provided tryptophan only in trace amount, and is therefore classified as near-LEAA (Supplementary Table S1).

Evidence from leucine-containing nutritional strategies without structured exercise is also informative, but should be interpreted cautiously. The PROVIDE trial enrolled 380 sarcopenic adults aged 65 years or older and compared a twice-daily oral nutritional supplement containing whey protein, leucine, and vitamin D with an isocaloric control for 13 weeks. The primary endpoints, handgrip strength and SPPB, did not reach statistical significance, although secondary outcomes including chair-stand performance and appendicular muscle mass improved [54]. Secondary analyses suggested that participants with sufficient baseline vitamin D status and protein intake experienced greater gains in muscle mass, and inflammatory markers such as IL-6 and IL-8 were reduced [48,49]. These findings are supportive of leucine-containing multimodal nutrition, but the formulation was not an LEAA mixture and the primary endpoints were negative.

Consistent with these observations, systematic reviews and meta-analyses suggest that leucine, leucine-enriched proteins, and BCAA interventions, often combined with vitamin D or exercise, may improve lean mass and selected functional outcomes in sarcopenia and related conditions [44,45]. However, because these analyses include heterogeneous formulations and populations, they should be regarded as supportive context rather than definitive evidence for LEAA-specific efficacy.

#### 4.2.3. Post-Exercise Recovery in Young and Active Populations

Evidence base: direct LEAA, 4 studies; near-LEAA, 2; free leucine, 1 (Table 2). The direct LEAA studies are small (*n* = 9–20) and short (4–8 days), and all reported surrogate outcomes rather than performance, injury, or other clinically meaningful endpoints. Two of the most frequently cited studies in this area used near-LEAA formulations containing 35% leucine [6,56].

Several short-term studies in young or physically active populations report effects of LEAA or near-LEAA formulations on surrogate markers of muscle damage, soreness, and inflammation. No study in this population evaluated performance, injury incidence, or other clinical endpoints. In a crossover study of healthy young adults, ingestion of 10 g of an EAA mixture enriched to 35% leucine during moderate steady-state exercise increased post-exercise muscle protein synthesis and suppressed whole-body protein turnover compared with an isonitrogenous EAA mixture containing a lower leucine proportion [6]. Because the leucine content was below the operational threshold used in this review, this study is best interpreted as near-LEAA evidence.

Small randomized studies have also examined post-exercise recovery after resistance or eccentric exercise. In young men, supplementation with 3.6 g of LEAA three times daily for eight days after eccentric elbow flexor exercise reduced peak creatine kinase activity, while effects on myoglobin, muscle soreness, and maximal strength recovery were less consistent [17]. In wheelchair basketball athletes, 4 g of LEAA supplementation three times daily for five days (days 1–5, spanning match play and the 4-day recovery period) reduced IL-6 and significantly improved relative ratings of perceived soreness compared with placebo [19]. Separately, a transcriptomic study in basketball athletes reported that 28 days of leucine supplementation was associated with upregulation of cytoskeleton-related genes, although the clinical significance of these molecular changes remains uncertain [62]. Mechanistic studies further suggest that LEAA supplementation can augment exercise-induced mTORC1 signaling, although this does not always translate into clear increases in integrated myofibrillar protein synthesis during recovery [18,63].

Overall, available evidence in active and young populations supports the biological plausibility that leucine-enriched EAA formulations may enhance acute post-exercise anabolic responses and modestly attenuate selected markers of muscle damage or inflammation. However, most studies were small, short in duration, and focused on surrogate recovery outcomes rather than long-term performance, injury prevention, or clinically meaningful endpoints. Therefore, these findings should be interpreted as supportive mechanistic and recovery evidence rather than definitive evidence of sustained functional benefit.

#### 4.2.4. Perioperative and Rehabilitative Cancer Care

Evidence base: direct LEAA, 3 studies; free leucine, 2; leucine-enriched protein, 3 (Table 2). The direct LEAA evidence comprises one acute tracer study (*n* = 24) [43], one pilot randomized trial with five participants per arm (*n* = 10) [20], and one non-randomized propensity-matched cohort (*n* = 152) [64]. No randomized trial with a prespecified clinical primary endpoint has been reported. Evidence relating to cancer cachexia specifically remains preclinical or mechanistic; none of the tabulated clinical studies enrolled a predominantly cachectic population.

Cancer cachexia represents a severe form of anabolic resistance, driven by systemic inflammation, accelerated muscle breakdown, and impaired amino acid-induced anabolism. Preclinical studies support the biological plausibility of leucine-based interventions: leucine has been shown to restore mTORC1 activity and downregulate ubiquitin–proteasome and autophagy pathways [3], and a high-protein diet enriched with leucine and fish oil preserved muscle mass and contractile function in tumor-bearing cachectic mice [65]. Human tracer data also suggest potential relevance in cancer-related catabolism. In patients with advanced non-small-cell lung cancer, a 14 g EAA mixture enriched to 40% leucine acutely improved whole-body protein balance despite persistent inflammation [43]. However, as noted above, this finding reflects both high EAA provision and leucine enrichment, and does not isolate the independent effect of leucine.

Clinical intervention studies remain heterogeneous. In an 8-week randomized controlled trial in older male patients with gastrointestinal cancer undergoing chemotherapy, free leucine supplementation combined with nutritional counseling was associated with within-group increases in body weight and appendicular skeletal muscle mass. However, no significant between-group difference was detected, most participants were non-cachectic or pre-cachectic at baseline, and the intervention used free leucine rather than an LEAA mixture [66]. Therefore, this study should be interpreted as indirect, hypothesis-generating evidence.

More direct LEAA evidence comes from perioperative and rehabilitation settings. In post-gastrectomy cancer patients, 15 days of 3 g LEAA supplementation (1.2 g leucine) twice daily combined with moderate resistance exercise improved muscle strength and quality of life compared with standard care, although there was no significant between-group difference in muscle mass [20]. Similarly, in patients undergoing major hepato-pancreato-biliary surgery for malignancy, prehabilitation combining structured home exercise with peri-exercise 3 g LEAA improved serum albumin, muscle-to-fat ratio, and postoperative length of stay [64]. A 12-week multimodal program in advanced cancer patients using resistance exercise, dietary counseling, and leucine-enriched whey improved handgrip strength, although the primary endpoint, SPPB, did not differ significantly [55].

Evidence from related interventions in this indication has grown but remains inconsistent. A randomized, double-blind trial of 6 g/day isolated leucine for four weeks did not meet its primary endpoint in patients with head and neck cancer, although a modest gain in appendicular muscle mass index was observed in an elderly non-cancer comparator cohort [21]. Two non-randomized studies reported favorable body composition changes with leucine-enriched oral nutritional supplements: one compared a leucine-enriched formula with a standard supplement in 142 malnourished patients, of whom about one-third had cancer [22], and one stratified 29 patients undergoing chemoradiotherapy by adherence to a combined exercise and nutrition program [23]. Neither used a randomized design, and none of these preparations is an LEAA mixture.

Overall, LEAA and related leucine-enriched interventions appear most plausible as part of multimodal cancer care, particularly when combined with exercise and adequate nutrition. Larger trials are needed to determine whether these approaches improve cachexia-specific outcomes, treatment tolerance, hospitalization, or survival.

#### 4.2.5. Maintenance Hemodialysis

Evidence base: direct LEAA, 1 study; free leucine, 2 (Table 2). The single direct LEAA study is an open-label randomized trial in 37 patients over 12 weeks in which the prespecified primary endpoint, the SF-36 physical component summary score, was not met [24]. The findings described below therefore rest on secondary outcomes of a single small trial.

Protein energy wasting is common in CKD, particularly among patients receiving maintenance hemodialysis. Contributing factors include dialytic amino acid losses, chronic inflammation, metabolic acidosis, and reduced physical activity, all of which can promote muscle atrophy and functional decline [67]. Hemodialysis itself removes approximately 10–12 g of amino acids per session [68], providing a rationale for targeted amino acid supplementation. LEAA formulations may be attractive in this setting because they provide anabolic stimulation with a relatively small amino acid load, although clinical evidence remains limited.

A recent Korean pilot study evaluated 6 g/day leucine combined with intradialytic resistance exercise for 12 weeks in 22 hemodialysis patients without baseline sarcopenia. Improvements in muscle mass, strength, and physical function were observed in a responsive subgroup, particularly among older women with lower baseline grip strength, lower hemoglobin or hematocrit, and good exercise adherence [69]. Because this intervention used leucine rather than an LEAA mixture and lacked a conventional control group, the findings should be considered supportive but indirect evidence.

A further randomized pilot trial in 24 patients undergoing maintenance hemodialysis compared 6 g/day of free leucine plus exercise with exercise alone over 12 weeks. Responder rates for handgrip strength and gait speed were higher with supplementation and skeletal muscle index increased modestly in the intervention group only, but the trial was explicitly designed as a pilot and was not powered for between-group inference [25].

Direct LEAA evidence in hemodialysis is still sparse. In a randomized Japanese trial, 37 hemodialysis patients with locomotive syndrome received locomotion training with or without daily supplementation of 3 g LEAA (1.2 g leucine) plus 800 IU vitamin D for 12 weeks. The primary endpoint, SF-36 physical composite score, did not differ significantly between groups. However, the supplement group maintained mental health scores and improved two-step test performance; these were secondary outcomes of a trial whose primary endpoint was not met, and the supplement was co-administered with vitamin D and locomotion training [24].

Overall, amino acid supplementation in hemodialysis is mechanistically plausible, but current evidence for LEAA-specific benefit is preliminary. Future trials should focus on patients with protein energy wasting or sarcopenia, define background protein intake, align supplementation timing with dialysis-related amino acid losses, and evaluate clinically meaningful outcomes such as physical function, hospitalization, frailty progression, and quality of life.

#### 4.2.6. Orthopedic Rehabilitation

Evidence base: direct LEAA, 1 study; mixed EAA plus intact protein, 1 (Table 2).

In a recent RCT in 65 knee osteoarthritis outpatients, 12 g/day of a formulation containing all nine essential amino acids together with intact whey and soy protein (6:4) for 8 weeks modestly increased DEXA-measured muscle density and improved SF-36 quality-of-life scores versus no intervention, without apparent hepato-renal or glycemic safety concerns [27]. Because the leucine proportion was not reported and the formulation included intact proteins, this study should be considered supportive rather than definitive LEAA evidence.

The single direct LEAA study is a randomized crossover trial in convalescent orthopedic inpatients, in which 3 g of an amino acid preparation containing 40% leucine (1.2 g) was administered once daily after exercise therapy during two one-month periods separated by a one-week washout, with an equienergetic starch placebo [26]. Most enrolled participants were discharged before the second period and were therefore excluded; 30 completed both periods and were analyzed per protocol, so the analyzed sample represents patients with slower functional recovery.

Both prespecified primary outcomes—skeletal muscle mass and limb muscle strength—were not met: neither rectus femoris cross-sectional area nor knee extension or grip strength differed between the supplemented and placebo conditions. The prespecified secondary outcomes (motor domain Functional Independence Measure, timed up-and-go, and nutritional status) likewise did not differ. A significant between-group difference was reported for rectus femoris echo intensity, an ultrasound-derived index of intramuscular fat; this measure was not listed among the prespecified primary or secondary outcomes of the trial, and the finding should accordingly be regarded as exploratory.

One further qualification concerns the formulation. The source publication describes the preparation as a “BCAA” supplement and names only leucine, isoleucine, valine, and lysine; the full indispensable amino acid profile is not reported there. We classify it as a direct LEAA formulation on the basis of composition: the same nine-essential-amino-acid preparation containing approximately 40% leucine is documented in another trial included in this review [28]. We note explicitly that this classification rests on information not contained in the trial publication.

#### 4.2.7. Neurological Conditions

Evidence base: direct LEAA, 1 study; leucine-enriched BCAA, 2; selected EAA formulation, 1 (Table 2). The single direct LEAA study is an exploratory randomized trial in 35 patients with mild Alzheimer’s disease over 28 days in which the prespecified primary endpoint (NPI-12) was not met; an improvement was reported for one secondary endpoint (Frontal Assessment Battery) [28]. The proposed mechanisms discussed below derive from preclinical models and are hypothesis-generating.

The potential neuroprotective effects of BCAAs and essential amino acids have attracted increasing interest, but the clinical evidence remains preliminary and heterogeneous. BCAA supplementation has been discussed in traumatic brain injury [46] and leucine-enriched BCAA (0.4 g/kg body weight; 45.5% leucine) has been explored as adjunctive therapy in autism spectrum disorder, with preliminary behavioral improvements reported in selected pediatric patients [32]. These findings are relevant to leucine-enriched amino acid biology, but they do not constitute direct LEAA evidence.

In cognitive impairment, an exploratory RCT in patients with mild Alzheimer’s disease evaluated a nine-essential-amino-acid mixture taken twice daily for 28 days. The formulation used in that trial contained nine essential amino acids, with leucine accounting for approximately 40% of total amino acid content by weight (1.61 g of ~4.0 g amino acids per serving), and therefore meets the operational LEAA definition used in this review. Separately, intake of seven selected essential amino acids improved attention, cognitive flexibility, and psychosocial function in middle-aged and older adults [29], but this intervention was not a full nine-EAA LEAA formulation.

The mechanisms proposed for these effects, including competition with kynurenine for transport across the blood–brain barrier, are discussed in Section 3 and shown in Figure 1C. Preclinical studies also suggest that EAA supplementation may restore brain neurotransmitter pools in aged mice [58]. In epilepsy, BCAA supplementation has been explored as an adjunct to ketogenic therapy [33], and LEAA enhanced antiseizure effects in rat models [59]. Overall, neurological applications remain hypothesis-generating, and future trials should use clearly defined formulations and clinically validated endpoints.

### 4.3. Indications Without Direct LEAA Evidence

No study using a formulation meeting the operational LEAA definition has been reported in either of the two indications in this section. The evidence summarized here is presented because it is clinically substantial and mechanistically informative for leucine-sensitive anabolic pathways, and because leucine-enriched BCAA supplementation in cirrhosis is the only application of a leucine-enriched amino acid intervention that is supported by clinical practice guidelines. It cannot, however, be extrapolated to LEAA mixtures, which differ from BCAA formulations in providing the full complement of indispensable amino acid substrates required for net protein synthesis. Findings in this section are excluded from the LEAA-specific synthesis in Section 4.4 and from the conclusions of this review.

#### 4.3.1. Liver Cirrhosis: Evidence from Leucine-Enriched BCAA

Evidence base: leucine-enriched BCAA, 9 studies; direct LEAA, none (Table 2). This is the most extensively investigated field in the leucine-enriched amino acid literature and the only one addressed by clinical practice guidelines, but no trial of a full-spectrum LEAA mixture has been conducted in cirrhosis. The guideline recommendations summarized below apply to BCAA supplementation and do not specify leucine enrichment as a requirement.

Leucine-enriched BCAA supplementation has been studied extensively in liver disease and represents one of the most clinically developed areas of leucine-enriched amino acid intervention. In a multicenter RCT involving 646 patients (622 analyzed) with decompensated cirrhosis, oral supplementation with 12 g leucine-enriched BCAA (leucine/isoleucine/valine = 2:1:1.2; 5.7 g leucine/day) significantly improved event-free survival, serum albumin levels, and quality of life (QOL) compared with diet therapy alone [41]. However, because BCAA formulations do not provide all nine indispensable amino acids, this evidence should be interpreted as clinically important leucine-enriched BCAA evidence rather than direct LEAA evidence.

Oral BCAA supplementation is recommended in several clinical practice guidelines. The 2020 Japanese Society of Gastroenterology cirrhosis guidelines recommend BCAA-enriched enteral nutrition as part of dietary management, oral BCAA granules for patients with hypoalbuminemia (serum albumin < 3.5 g/dL), ascites, or hepatic encephalopathy, and regular nutritional assessment in patients at risk of protein energy malnutrition or sarcopenia [70]. The EASL and ESPEN guidelines make comparable recommendations [71,72,73]; the ESPEN practical guideline advises long-term oral BCAA supplementation at 0.25 g/kg/day in advanced cirrhosis to improve event-free survival or quality of life (Recommendation 60, Grade B).

These guidelines support oral BCAA supplementation as part of nutritional management in cirrhosis, particularly in patients with protein energy malnutrition, hypoalbuminemia, ascites, hepatic encephalopathy, or sarcopenia risk. In Japan, the most widely studied preparation is an oral BCAA granule formulation with a leucine/isoleucine/valine ratio of 2:1:1.2, corresponding to approximately 48% leucine [41,42]. This high leucine proportion is mechanistically relevant to anabolic signaling, but the guideline recommendations apply to BCAA supplementation in general and do not specify leucine enrichment as a requirement.

The potential benefits of BCAA supplementation have also been examined in cirrhosis-associated sarcopenia. Patients with cirrhosis frequently experience reduced muscle mass, muscle quality, and strength, which are associated with poor clinical outcomes. Continuous BCAA supplementation, typically approximately 5–11 g/day of leucine-enriched BCAA administered for at least one month, has been associated with improvements in muscle mass or sarcopenia-related parameters in several studies [34,35,36,37]. These effects may be partly explained by restoration of impaired mTORC1 signaling and attenuation of autophagy-related pathways, as suggested by mechanistic data in alcoholic cirrhosis [51]. Nevertheless, the clinical response appears heterogeneous and may depend on disease severity, baseline nutritional status, protein intake, exercise, and concomitant standard care.

In a retrospective study, sarcopenia was prevalent and associated with higher mortality, while long-term BCAA supplementation at 12 g/day (5.7 g leucine/day for ≥1 year) was associated with improved survival among sarcopenic adults with cirrhosis [42]. However, two randomized studies did not demonstrate clear benefits of BCAA supplementation on sarcopenia-related outcomes [38,39]. Potential explanations include differences in control-group protein intake, dietary counseling, baseline nutritional status, exercise exposure, disease stage, and the use of other nutritional supplements. Therefore, although BCAA supplementation is guideline-supported in cirrhosis and has biologically plausible effects on muscle metabolism, its impact on sarcopenia outcomes is not uniform across studies.

A single-arm pilot study combined supervised home-based exercise with a BCAA-enriched supplement in 12 patients with compensated cirrhosis and sarcopenia, reporting substantial within-patient increases in skeletal muscle index and improvement in the Liver Frailty Index over 12 weeks [40]. The absence of a control group precludes attribution to either component, and the study again used a BCAA rather than an LEAA preparation.

For the purpose of this review, liver disease evidence should therefore be regarded as strong adjacent evidence for leucine-enriched amino acid intervention, but not as direct evidence for full-spectrum LEAA mixtures. Future studies should clarify whether combining leucine-enriched BCAA with a full-spectrum EAA formulation provides additive benefits, since EAA mixtures supply all substrates required for net protein synthesis whereas BCAA alone cannot sustain protein synthesis without adequate availability of other indispensable amino acids [43,50]. Trials should also stratify participants by disease severity and nutritional status, define protein intake in both intervention and control groups, optimize dose timing, including use as a late-evening snack, which is standard practice in cirrhosis, and incorporate exercise or rehabilitation where feasible.

#### 4.3.2. Heart Failure and Other Emerging Areas

Evidence base: near-LEAA, 1 study; direct LEAA, none (Table 2). The single tabulated study used a near-LEAA formulation containing 34% leucine in an acute crossover design with eight participants [74]; the accompanying systematic review [47] covered heterogeneous protein and essential amino acid preparations rather than LEAA mixtures.

In heart failure, a systematic review of six trials and a crossover RCT in older women suggested that essential amino acid supplementation at doses ranging from 3 to 15 g/day may modestly improve 6 min walk distance, lean body mass, or whole-body protein balance [47,74]. These findings are biologically plausible, but the evidence base remains small and heterogeneous.

Evidence in this area is preliminary and does not permit LEAA-specific conclusions.

### 4.4. Synthesis Restricted to Formulations Meeting the LEAA Definition

This section synthesizes only the 19 studies that used formulations meeting the operational LEAA definition (Table 2). Evidence from near-LEAA, leucine-enriched BCAA, free leucine, leucine-enriched protein, and selected EAA formulations is excluded here, irrespective of its size or clinical importance.

Acute stimulation of muscle protein synthesis. Five direct LEAA studies used stable-isotope tracer or intracellular signaling endpoints [4,9,10,43,63]. These provide the most internally consistent finding in the review: in older adults, LEAA doses as low as 1.5–3 g stimulated myofibrillar protein synthesis to a degree comparable with substantially larger whey protein boluses [9,10], and a mixture containing 41% leucine increased fractional synthesis rate where an isonitrogenous 26% leucine mixture did not [4]. Two qualifications apply. Each of these studies enrolled 8–24 participants, and in the one study conducted in a disease population the authors attributed the anabolic advantage primarily to total essential amino acid content rather than to leucine enrichment per se [43].

Dose. Across the direct LEAA studies, the doses at which effects were observed were consistently low. Stimulation of muscle protein synthesis was demonstrated at 1.5 g in a dose-ranging study [10] and at 3 g in older women, in both cases to a degree comparable with 20–40 g of whey protein [9,10]; the largest acute dose tested was 6.7 g [4]. Chronic trials used 3–6 g/day in most cases, with a maximum of 22 g/day of a near-LEAA mixture [14]. No study has identified an upper dose beyond which the response plateaus, and no formal dose ranging trial has been conducted over a clinically relevant duration. Within the range studied, however, the amino acid load required to elicit an anabolic response is an order of magnitude smaller than the protein bolus conventionally recommended for older adults, and this remains the most distinctive practical property of these formulations.

Functional and body composition outcomes. Fourteen direct LEAA studies reported clinical or functional outcomes over periods from 4 days to 6 months [11,12,13,15,16,17,18,19,20,24,26,28,30,64]. Their results are not uniform. A prespecified primary endpoint was explicitly reported and met in two trials [15,30]; was explicitly reported and not met in six [11,13,16,24,26,28]; and was not clearly prespecified or was reported without a stated hierarchy in the remainder. Twelve of the fourteen administered LEAA together with exercise, rehabilitation, vitamin D, or dietary counseling, so that the independent effect of LEAA cannot be isolated. No direct LEAA trial has evaluated hospitalization, treatment tolerance, frailty progression, or survival.

Of the 19 studies meeting the operational LEAA definition, most were exploratory in design (Supplementary Table S1): 10 enrolled fewer than 30 participants, and a prespecified primary endpoint was explicitly reported in eight. This distribution reflects the early stage of formulation-specific clinical development rather than inconsistency of findings.

What the direct evidence supports. Taken together, the direct LEAA evidence supports the conclusion that low-dose, leucine-enriched essential amino acid mixtures acutely stimulate muscle protein synthesis in older adults with an efficiency disproportionate to their amino acid load. A related observation supports the compositional rationale rather than qualifying it. In the one trial that isolated leucine from its substrate—adding 7.5 g/day of free leucine to an already optimized protein intake—no additional anabolic effect was observed [5], whereas leucine proportion determined the response when total amino acid content was held constant [4]. This pattern is consistent with the interpretation advanced in Section 3: leucine enrichment raises the anabolic efficiency of a defined amino acid load, but does not substitute for, and does not add to, an already sufficient supply of indispensable amino acids. The direct LEAA evidence does not currently support conclusions about sustained improvement in strength, mobility, quality of life, or any patient-important clinical outcome, either for LEAA alone or, given the absence of LEAA-only comparator arms, for LEAA as an additive component of multimodal programs.

## 5. Safety and Tolerability

Safety was not a prespecified focus of most studies in this field, and the evidence summarized here should be read as a description of what has been reported rather than as a systematic safety evaluation. Of the 50 human studies cataloged in Supplementary Table S1, eight reported adverse events systematically, with prespecified collection and reporting; thirteen mentioned adverse events without a systematic reporting framework; and twenty-nine did not report adverse events at all. Laboratory safety parameters were reported in eight studies and explicitly not assessed in one.

Where adverse events were reported, they were predominantly gastrointestinal or related to palatability. In a 12-month randomized trial in cirrhosis, distaste was significantly more frequent with leucine-enriched BCAA than with a whey protein comparator [39]. In knee osteoarthritis, no hepatic, renal, or glycemic safety concerns were identified over 8 weeks, with 93% adherence [27]. In a six-month randomized trial of LEAA with exercise in older adults, adverse events were collected prospectively and renal function was monitored, with no serious events reported and renal function unchanged [16]. In a crossover trial in convalescent orthopedic inpatients, adverse events were prespecified and renal function was measured before and after each period, with no events attributable to the intervention [26]. No study reported a safety signal attributable to the amino acid intervention that led to trial discontinuation.

Three limitations of this evidence base should be emphasized. First, the duration of exposure across the tabulated studies was short: only three of the fifty studies followed participants for twelve months or longer [39,41,42], so the long-term safety of sustained leucine-enriched amino acid supplementation is essentially uncharacterized. The exception is a two-year cirrhosis trial in which increases in serum ALT and AST were recorded alongside gastrointestinal complaints [41]; this trial nevertheless represents the largest and longest safety exposure available for any leucine-enriched amino acid preparation, and no signal led to its termination. Second, the populations in which safety data would be most consequential—patients with advanced cirrhosis, chronic kidney disease receiving hemodialysis, and active malignancy—are represented by small studies in which adverse events were reported inconsistently. In hemodialysis in particular, direct LEAA evidence comprises a single trial of 37 patients over 12 weeks [24]. Third, dose-related tolerability has not been formally examined; the only dose ranging study identified compared 1.5 g and 6 g LEAA in an acute setting and did not report tolerability outcomes [10].

Accordingly, we do not conclude that LEAA mixtures are established as safe. Available studies have not reported clear safety signals, but safety has not been systematically evaluated and follow-up has generally been limited. It is also worth noting what the reported events were not. The events that recurred were gastrointestinal symptoms and unpalatability, and these were the stated reasons for withdrawal in three of the longer supplementation studies [11,36,69]. Tolerability in this field has therefore been constrained less by toxicity than by the volume and palatability of the preparations used, which is the practical problem that low-dose formulations are intended to address. Prospective collection of adverse events, discontinuations, and laboratory safety parameters should be a standard component of future trials, particularly in renal and hepatic populations.

## 6. Limitations

Several limitations should be considered when interpreting this review. First, the operational boundary between LEAA mixtures, leucine-enriched BCAA, free leucine, and leucine-enriched protein formulations is not uniformly defined in the literature. Although this review defined LEAAs as mixtures containing all nine indispensable amino acids with approximately 40% or more leucine by weight, some included studies used borderline formulations, such as 35% leucine EAA [6], or interventions outside this definition, such as leucine-enriched whey protein [61], free leucine alone [66], or leucine-enriched BCAA in liver disease. We labeled these intervention types separately, but comparisons across categories remain limited by compositional heterogeneity. The operational threshold of approximately 40% leucine is a classification convention adopted for this review and should not be read as a validated biological cut-off. Studies falling on either side of it may not differ meaningfully in their physiological effect, and the classification is further sensitive to how the leucine fraction is expressed: in one trial the preparation provided leucine at 37.9% of total amino acid content but 41.6% of the nine indispensable amino acids [7].

Second, the independent contribution of leucine enrichment is not always distinguishable from the effect of total EAA provision. For example, Engelen et al. [43] reported greater net anabolism with a leucine-enriched EAA mixture in advanced cancer patients, but attributed the advantage mainly to higher EAA content rather than leucine enrichment per se. This distinction is central to interpreting LEAA mechanisms and requires further formulation-controlled studies.

Third, this was a narrative rather than a systematic review or meta-analysis. Therefore, study selection and emphasis may reflect author judgment, and the review may be subject to selection or reporting bias. Although the search strategies are now fully documented and their coverage of the tabulated studies has been measured (Supplementary Methods S1), study screening and selection were not recorded prospectively and were not performed in duplicate; no screening log or record-level exclusion count is available. Selection of studies for discussion therefore reflects author judgment applied to the eligibility considerations set out in Section 2.3, and this limitation is not fully remedied by retrospective documentation of the search. In addition, many available studies were small, short in duration, and heterogeneous in population, formulation, dose, timing, comparator, and co-interventions such as exercise, vitamin D, dietary counseling, or rehabilitation. Several studies also reported benefits mainly in secondary endpoints, within-group changes, exploratory analyses, or selected subgroups. These features limit causal attribution and generalizability. Larger, longer, formulation-specific trials with predefined primary endpoints and clinically meaningful outcomes are needed to clarify optimal dosing, timing, target populations, and long-term benefits.

## 7. Conclusions

The conclusions below come from Section 4.4, which covers only formulations that meet the operational LEAA definition. LEAA mixtures combine a leucine signal that activates anabolic signaling with the indispensable amino acids needed to sustain it. Direct human evidence supports this in one respect: in acute tracer studies in older adults, 1.5–3 g of a mixture containing about 40% leucine promoted muscle protein synthesis as much as far larger doses of whey protein [9,10], and it was the leucine proportion, not the total amino acid content, that determined the response [4]. Adding free leucine to a diet already sufficient in protein gave no further benefit [5], which suggests that the efficiency lies in the combination and not in leucine alone. In practical terms, the same anabolic stimulus as a large protein serving can be given in a few grams.

Whether that efficiency leads to lasting clinical benefit is not yet known. The studies with functional or body composition outcomes were mostly exploratory, and almost all gave LEAA alongside another intervention; so, the effect of LEAA on its own cannot be separated. None looked at hospitalization, treatment tolerance, frailty progression, or survival, and safety has not been assessed systematically (Section 4.4 and Section 5). Evidence from related interventions (Section 4.3) is not used to fill these gaps.

The evidence gathered here does, however, show where the next trial should be conducted. Direct LEAA evidence is concentrated in older adults, where the acute response is consistent and the small dose is most likely to matter, yet the longest exposure reported is six months. What is needed is a trial of a clearly defined LEAA formulation against an isonitrogenous comparator, with protein intake and activity kept the same in both groups, a prespecified functional primary endpoint, and safety data collected prospectively for at least six months. Such a trial would matter most for patients who cannot easily eat more protein, such as those with poor appetite, early satiety, swallowing difficulty, or fluid and protein limits. A small dose may also help them keep taking it, since the volume and taste of larger supplements have been a common reason for dropping out [11,62,69]. A trial on this scale is realistic. In cirrhosis, a leucine-enriched amino acid preparation entered clinical practice guidelines based on the strength of a multicenter randomized trial of 646 patients followed for two years to an event-free survival endpoint [41]. That trial says nothing about LEAA, but it shows the standard that LEAA formulations have yet to meet.

LEAAs are best described as compact, mechanistically grounded preparations with a proven acute anabolic effect in older adults. The task now is to find out whether that effect brings benefits that patients can feel, in the people who need it most.

## Figures and Tables

**Table 1:** Summary of findings from clinical studies of LEAA mixtures and related leucine-enriched amino acid interventions, by clinical application.

Clinical Application	Stratification	Evidence Category	Doses Tested	Duration	Summary of Findings	Co-Inter-Ventions	Refs.
Muscle protein synthesis in older adults	Older adults	Direct LEAA *; free leucine	1.5–6.7 g; 7.5 g/d	2–6 h; 12 wk	In acute tracer studies, 1.5–3 g LEAA increased muscle protein synthesis to an extent comparable with 20–40 g whey protein (between-group; *n* = 16 and *n* = 24) [9,10]. A 41% leucine mixture increased fractional synthesis rate whereas an isonitrogenous 26% leucine mixture did not; younger participants responded to both (*n* = 36; non-randomized) [4]. Adding 7.5 g/d free leucine to resistance training in older women whose protein intake was already optimized produced no further increase in myofibrillar fractional synthesis rate versus alanine (*n* = 19) [5], indicating that the response depends on the combination of leucine and indispensable amino acid substrate rather than on leucine alone.	Resistance exercise; protein optimization	[4,5,9,10]
Body composition and physical performance	Older adults	Direct LEAA; one near-LEAA	3–22 g/d; 0.21 g/kg/d	12 wk–6 mo	Lean tissue mass increased from baseline in the 40% leucine group (1.1%; within-group), and functional performance improved from baseline in both amino acid groups, but no between-group difference was observed for either primary outcome, and no additional effect was seen with 40% versus 20% leucine (pilot; 25 of 36 analyzed) [11]. Walking measures improved versus exercise alone at 6 months (between-group; *n* = 29) [12]. In a placebo-controlled trial with exercise in both arms, the primary endpoints (muscle mass, strength) were not met; low-back discomfort improved as a secondary endpoint (*n* = 130) [13]. A single-arm near-LEAA study reported within-group gains in lean mass and leg strength (*n* = 12; no control) [14].	Exercise; arginine	[11,12,13,14]
Sarcopenia and exercise combination care	Older adults	Direct LEAA; near-LEAA	3–6 g/d; 11 g/d	3–6 mo	With resistance and balance training, knee extension strength, gait speed and appendicular lean mass improved versus control (between-group; *n* = 155; endpoint hierarchy not stated); LEAA alone improved walking speed [15]. In a 6-month open-label trial in which both arms received LEAA, the prespecified primary outcome (appendicular skeletal muscle mass index) was not met in either arm; gait speed improved in both, without a between-group difference (*n* = 66) [16]. A four-arm trial found greater gains in muscle mass and functional test scores with exercise plus a near-LEAA mixture than with either alone (*n* = 96) [7].	Resistance and balance training	[7,15,16]
Post-exercise recovery care	Young men	Direct LEAA	10.8–12 g/d	4–8 d	Peak creatine kinase was lower than placebo, while myoglobin, soreness and strength recovery were inconsistent (crossover; *n* = 10) [17]. Total peak torque was better preserved and soreness lower over 96 h, without a difference in integrated myofibrillar protein synthesis (parallel; *n* = 20) [18]. Men only; surrogate endpoints.	Eccentric or resistance exercise	[17,18]
Post-exercise recovery care	Athletes	Direct LEAA	12 g/d	5 d	IL-6 and relative ratings of perceived soreness were lower than placebo; creatine kinase and TNF-α did not differ (crossover; *n* = 9; wheelchair basketball players) [19].	Match play and recovery	[19]
Cancer care, perioperative and rehabilitative	Cancer patients	Direct LEAA; free leucine; leucine-enriched protein	6 g/d; 6 g/d; ONS providing 3–4.6 g leucine/d	15 d–3 mo	With resistance training, lower-limb strength and physical and mental QOL scores improved versus standard care; no between-group difference in muscle mass (open-label pilot; five per arm) [20]. A randomized, double-blind trial of 6 g/d isolated leucine did not meet its primary endpoint (appendicular skeletal muscle mass index) in head and neck cancer (*n* = 41) [21]. Two non-randomized studies of leucine-enriched oral nutritional supplements reported favorable body composition changes (*n* = 142 [22]; *n* = 29, adherence-stratified [23]).	Resistance exercise; nutrition counseling	[20,21,22,23]
Renal disease care, hemodialysis	Dialysis patients	Direct LEAA; free leucine	3 g/d; 6 g/d	3 mo	The primary endpoint (SF-36 physical component summary) was not met; mental-health scores were maintained and two-step test performance improved as secondary outcomes (open-label; *n* = 37) [24]. A randomized pilot of 6 g/d free leucine plus exercise reported higher responder rates for handgrip strength and gait speed but was not powered for between-group inference (*n* = 24) [25].	Vitamin D; locomotion training; exercise	[24,25]
Orthopedic rehabilitation	Convalescent inpatients	Direct LEAA	3 g/d	1 mo per period (crossover)	Both prespecified primary outcomes (skeletal muscle mass, limb muscle strength) were not met, neither were the prespecified secondary outcomes (FIM, timed up-and-go, nutritional status). Rectus femoris echo intensity improved relative to placebo, but this was not a prespecified outcome (crossover; 30 analyzed per protocol from a larger enrolled cohort) [26].	Exercise therapy	[26]
Knee osteoarthritis	Outpatients	Mixed EAA plus intact protein	12 g/d	8 wk	DEXA-measured muscle density and SF-36 scores improved versus a no-intervention control; WOMAC improved but not versus control (*n* = 65). The formulation contains intact whey and soy protein and its leucine fraction was not reported [27].	None specified	[27]
Neurological care, cognitive impairment	Mild Alzheimer’s disease; middle-aged and older adults	Direct LEAA; selected EAA	8 g/d; 3 or 6 g/d	28 d; 12 wk	The primary endpoint (NPI-12) was not met; FAB improved as a secondary endpoint by ANCOVA (*n* = 35) [28]. A seven-EAA formulation improved Trail Making Test B, social interaction and psychological scores in the 6 g/day arm only (multiplicity-adjusted; *n* = 103) [29].	None specified	[28,29]
Neurological care, stroke-related	Post-stroke patients	Direct LEAA; leucine-enriched BCAA	3 g/d; 12 g/d	8 wk; 1 mo	With low-intensity resistance training, the primary endpoint (motor-domain FIM) was met versus control (between-group difference 9.2; open-label with blinded assessment; *n* = 44) [30]. With comprehensive rehabilitation, grip strength and skeletal muscle index improved more than in a matched retrospective control (non-randomized; *n* = 54; BCAA) [31].	Resistance training; rehabilitation	[30,31]
Neurological care, neurodevelopmental disorder	Children	Leucine-enriched BCAA	0.4 g/kg/d	>10 wk	In an open-label uncontrolled pilot, 26 of 55 children (47%) who continued beyond 10 weeks were reported to show improvements in social behavior, speech and hyperactivity. No control group [32].	None specified	[32]
Neurological care, seizure disorders	Children on ketogenic diet	Leucine-enriched BCAA	up to 20 g/d	2–12 mo	In an uncontrolled pilot in 17 children, seizures were reported to cease in 3 and to fall by 50–90% in 5, while the ketogenic diet ratio was concurrently changed from 4:1 to 2.5:1. No control group [33].	Ketogenic diet	[33]
Liver cirrhosis, muscle outcomes	Cirrhotic patients	Leucine-enriched BCAA	5–21 g/d	1–12 mo	Muscle mass or sarcopenia-related measures improved versus control in four studies [34,35,36,37]. Two randomized trials did not meet their primary endpoints: change in CT-based skeletal muscle index [38], and a co-primary of at least 5% increase in grip strength and/or upper-limb lean mass at 12 months versus an equicaloric, equinitrogenous whey comparator [39]. A single-arm pilot reported large within-patient increases in skeletal muscle index and Liver Frailty Index without a control group (*n* = 12) [40]. No LEAA mixture has been studied in this population.	Exercise; diet counseling	[34,35,36,37,38,39,40]
Liver cirrhosis, survival and QOL	Cirrhotic patients	Leucine-enriched BCAA	7.2–12 g/d	16 wk–2 y	Event-free survival, serum albumin and the SF-36 general health perception score improved versus diet alone (primary endpoint met; open-label; 622 analyzed) [41]. Cirrhosis-related complications were fewer and event-free survival better in an open-label trial with an active comparator [36]. Liver Frailty Index and albumin improved [37]. Lower mortality was associated with BCAA in a retrospective cohort [42]. No LEAA mixture has been studied in this population.	Diet therapy	[36,37,41,42]

Abbreviations of units: h, hour; d, day; wk, week; mo, month; y, year. Evidence categories are as defined in Section 2.4 and tabulated in Table 2. Findings are stated as reported in the source publications; “between-group” denotes a statistically significant difference between randomized groups, and secondary endpoints, within-group changes, and unmet primary endpoints are identified as such. Doses are those of the amino acid preparation, not of leucine alone. This table summarizes representative studies for each clinical application; the complete catalog of the 50 tabulated studies, with prespecified primary endpoints, principal limitations, safety information, and design and endpoint descriptors, is given in Supplementary Table S1, and the distribution of intervention categories across indications is shown in Table 2. * Some trials listed under “Direct LEAA” included a lower-leucine essential amino acid comparator arm that does not meet the operational LEAA threshold (approximately 40% leucine); e.g., a 26% leucine arm in Katsanos et al. [4] and a 20% leucine arm in Ispoglou et al. [11]. In these studies, only the higher leucine arm qualifies as LEAA, and the table reflects that arm. Børsheim et al. [14] used a single-arm near-LEAA mixture (approximately 36% leucine) and is included for clinical context. Ispoglou et al. [11] dosed by body weight (0.21 g/kg/day) rather than as a fixed daily amount.

**Table 2:** Evidence map: distribution of study types across clinical indications.

Clinical Indication (Section)	Direct LEAA	Near-LEAA	Leu-Enr. BCAA	Free Leucine	Leu-Enr. Protein	Selected EAA/Mixed	Total	Nature and Adequacy of Direct LEAA Evidence
Indications with direct LEAA evidence (Section 4.2)								
Muscle protein synthesis and body composition in older adults (Section 4.2.1)	4	1	–	2	1	–	8	Three acute tracer studies (*n* = 16–24) consistently positive: 1.5–3 g stimulated muscle protein synthesis comparably to 20–40 g whey protein, and leucine proportion rather than nitrogen content determined the response. One 12-week pilot RCT (25 of 36 completed); longest direct LEAA exposure, 12 weeks. Free leucine added to an already optimized protein intake conferred no further benefit, consistent with the signal-plus-substrate rationale. The doses at which effects were observed (1.5–3 g) are an order of magnitude below the protein bolus conventionally recommended for older adults.
LEAA with exercise or rehabilitation in older adults (Section 4.2.2)	5	1	1	–	1	–	8	Five RCTs (*n* = 29–155, 3–6 months), the largest body of controlled evidence for any indication. Prespecified primary endpoint met in two, including gains in strength, gait speed and lean mass with training, and in motor domain functional independence after stroke; not met in two; not clearly prespecified in one. All evaluated LEAA together with exercise and none included an LEAA-only arm, so the independent LEAA effect is not isolable.
Post-exercise recovery in young and active populations (Section 4.2.3)	4	2	–	1	–	–	7	Four small short-term RCTs (*n* = 9–20; 4–8 days). Outcomes limited to surrogate markers (creatine kinase, soreness, IL-6, intracellular signaling); no clinical endpoints.
Perioperative and rehabilitative cancer care (Section 4.2.4)	3	–	–	2	3	–	8	One acute tracer study (*n* = 24), one pilot RCT (*n* = 10), one propensity-matched cohort (*n* = 152). No RCT with a prespecified clinical primary endpoint. A randomized trial of free leucine did not meet its primary endpoint in this population.
Maintenance hemodialysis (Section 4.2.5)	1	–	–	2	–	–	3	Single open-label RCT of LEAA (*n* = 37, 12 weeks); prespecified primary endpoint (SF-36 physical component) not met. Two free-leucine studies, both pilot in scale.
Orthopedic rehabilitation (Section 4.2.6)	1	–	–	–	–	1	2	Single crossover RCT (30 analyzed per protocol); both prespecified primary outcomes (muscle mass, limb strength) not met; the significant finding (echo intensity) was not a prespecified outcome.
Neurological conditions (Section 4.2.7)	1	–	2	–	–	1	4	Single exploratory RCT (*n* = 35, 28 days); prespecified primary endpoint (NPI-12) not met; benefit reported for one secondary outcome (FAB).
Indications without direct LEAA evidence (Section 4.3)								
Liver cirrhosis (Section 4.3.1)	0	–	9	–	–	–	9	None. No trial of a full-spectrum LEAA mixture has been reported. All evidence, including that underpinning clinical practice guidelines, derives from leucine-enriched BCAA.
Heart failure (Section 4.3.2)	0	1	–	–	–	–	1	None. The single tabulated study used a near-LEAA formulation (34% leucine) in an acute crossover design (*n* = 8).
Total	19	5	12	7	5	2	50	

Counts refer to the 50 human studies cataloged in Supplementary Table S1 and are assigned by the amino acid preparation actually administered, as defined in Section 2.4. Each study is counted once, under the indication in which it is principally discussed; studies contributing to more than one section (e.g., Engelen et al. [43], discussed in both Section 3 and Section 4.2.4) are counted under the latter only. Systematic reviews and meta-analyses [44,45,46,47], secondary analyses of previously tabulated trials [48,49], and mechanistic human studies discussed only in Section 3 [50,51,52] are not counted. Studies including both a qualifying (approximately 40% leucine) arm and a lower-leucine comparator arm [4,11] are counted as direct LEAA. “Selected EAA/mixed” comprises one seven-EAA formulation [29] and one EAA-plus-intact-protein formulation whose leucine fraction was not reported [27]. The final column summarizes the design, size, and endpoint status of the direct LEAA studies only; it is intended as a transparent indication of evidence adequacy and does not constitute a formal risk-of-bias or certainty-of-evidence assessment. Detailed study characteristics, including prespecified primary endpoints, principal limitations, safety information, and design and endpoint descriptors, are provided in Supplementary Table S1.

## Data Availability

No new data were created or analyzed in this study. Data sharing is not applicable to this article.

## Supplementary Materials

The following supporting information can be downloaded at: https://www.mdpi.com/article/10.3390/nu18183024/s1, Supplementary Methods S1: Search strategies, records retrieved, and identification routes for the 50 tabulated studies, including the complete Boolean strings for the intervention block and all eight domain blocks; Supplementary Table S1: Characteristics of the 50 human studies discussed in this review, with population, intervention, duration, design, co-interventions, key findings, prespecified primary endpoint and whether it was met, principal limitations, safety and tolerability as reported, and design tier and endpoint level descriptors.

## Abbreviations

The following abbreviations are used in this manuscript:

LEAA	Leucine-enriched essential amino acid
EAA	Essential amino acid
BCAA	Branched-chain amino acid
mTORC1	Mammalian target of rapamycin complex 1
FOXO1	Forkhead box protein O1
MPS	Muscle protein synthesis
FSR	Fractional synthesis rate
CKD	Chronic kidney disease
RCT	Randomized controlled trial
QOL	Quality of life
DEXA	Dual-energy X-ray absorptiometry
ESPEN	European Society for Clinical Nutrition and Metabolism
EASL	European Association for the Study of the Liver
SPPB	Short Physical Performance Battery
FAB	Frontal Assessment Battery
SF-36	Short Form-36 Health Survey
NPI-12	Neuropsychiatric Inventory-12
FIM	Functional Independence Measure
ASMI	Appendicular skeletal muscle mass index
SMI	Skeletal muscle index
WOMAC	Western Ontario and McMaster Universities Osteoarthritis Index
PI3K	Phosphoinositide 3-kinase
Akt	Protein kinase B
LAT1	L-type amino acid transporter 1
S6K1	Ribosomal protein S6 kinase 1
4E-BP1	Eukaryotic translation initiation factor 4E-binding protein 1
MeSH	Medical Subject Headings
