# Supplementary material for: Leucine-Enriched Essential Amino Acids in Clinical Practice: A Narrative Review
Source: Nutrients. 2026 Sep 16;18(18):3024. doi: 10.3390/nu18183024 (PMC13610391; doi:10.3390/nu18183024)
Supplement: Supplementary file 1 [file nutrients-18-03024-s001.zip › nutrients-4523288-Supplementary_Methods_S1.pdf]

Supplementary Methods S1

Search strategies, records retrieved, and identification routes for the tabulated studies

Nagao K, Hasegawa T, Kihara H, Nishikata N, Kobayashi H, Atherton PJ, Fujita S. Leucine-Enriched Essential Amino Acids in Clinical Practice: A Narrative Review.

S1.1. Search strategy and records retrieved

PubMed (MEDLINE) and Google Scholar were searched on 3 September 2026, from database inception to that date; no separate update search was therefore required. Searches were constructed as a common intervention block combined in turn with each of eight domain blocks (Section S1.3). The intervention block alone returned 314,970 records. Species and language were not applied as search filters; restriction to human studies published in English was applied at the screening stage (Section 2.3 of the main text), because the PubMed species filter operates on assigned MeSH terms and would have excluded eight of the studies cited in this review for reasons unrelated to their content. Google Scholar does not return stable result counts, and none is reported for it.

Table S1-1. Records retrieved by each search, PubMed, 3 September 2026.

Search	Domain block combined with the intervention block	Records
A	Muscle protein synthesis and protein turnover	8,376
B	Sarcopenia, ageing, and body composition	3,844
C	Exercise and rehabilitation	2,439
D	Liver cirrhosis	2,858
E	Cancer and cachexia	25,465
F	Chronic kidney disease and haemodialysis	2,792
G	Neurological conditions	10,916
H	Heart failure and orthopaedic conditions	3,432
	**Sum of searches A–H**	**60,122**
	**Unique records after removal of overlap**	**54,063**

S1.2. Identification routes for the tabulated studies

Each of the 50 studies catalogued in Supplementary Table S1 was retrieved by digital object identifier and intersected with the combined search result. Forty-nine are indexed in PubMed; the exception is Kitabayashi et al. [24], published in a journal not indexed for MEDLINE. Of the 49 indexed studies, 47 (96%) were retrieved by the search strategy.

The two exceptions failed at different points, and both reflect a structural limitation of term-based retrieval rather than a defect in the strings used. Nakajima et al. [64] does not name the amino acid preparation in its title, abstract, or assigned MeSH terms, describing the intervention only as preoperative nutritional therapy, and therefore does not satisfy the intervention block. Wang et al. [62] satisfies the intervention block but reports transcriptomic and athletic-performance outcomes that fall outside all eight domain blocks.

Accordingly, 47 of the 50 tabulated studies were identified through the database searches and three

[24,62,64] from the authors' prior knowledge of the field. We report this figure not to claim completeness, but to allow readers to establish how far the documented strategy accounts for the evidence discussed.

Screening and selection were not recorded prospectively: no screening log, duplicate assessment, or record-level exclusion count is available. The number of records retrieved is reported so that the searches can be reproduced, not to imply that all retrieved records were formally screened against predetermined criteria. This limitation is also stated in Section 6 of the main text.

S1.3. Boolean search strings

Reproduced exactly as entered in PubMed. Each domain block was combined with the intervention block using AND. No species or language filter was applied.

Intervention block

```
("leucine"[MeSH Terms] OR "leucine"[tiab] OR "leucine-enriched"[tiab] OR
"amino acids, essential"[MeSH Terms] OR "essential amino acid*"[tiab] OR
"EAA"[tiab] OR "LEAA"[tiab] OR "amino acids, branched-chain"[MeSH Terms] OR
"branched-chain amino acid*"[tiab] OR "BCAA"[tiab])
```

A — Muscle protein synthesis and protein turnover

```
("muscle proteins"[MeSH Terms] OR "muscle protein synthesis"[tiab] OR
"protein turnover"[tiab] OR "myofibrillar protein synthesis"[tiab] OR
"fractional synthesis rate"[tiab] OR "TOR Serine-Threonine Kinases"[MeSH Terms] OR
"mTORC1"[tiab] OR "anabolic resistance"[tiab] OR "muscle protein breakdown"[tiab])
```

B — Sarcopenia, ageing, and body composition

```
("Sarcopenia"[MeSH Terms] OR sarcopeni*[tiab] OR "Frailty"[MeSH Terms] OR
frailty[tiab] OR "Muscular Atrophy"[MeSH Terms] OR "muscle wasting"[tiab] OR
"older adult*"[tiab] OR elderly[tiab] OR "Body Composition"[MeSH Terms] OR
"physical function*"[tiab] OR "Muscle Strength"[MeSH Terms])
```

C — Exercise and rehabilitation

```
("Exercise"[MeSH Terms] OR "Resistance Training"[MeSH Terms] OR
"exercise recovery"[tiab] OR "muscle damage"[tiab] OR "muscle soreness"[tiab] OR
"delayed onset muscle soreness"[tiab] OR "Rehabilitation"[MeSH Terms] OR
rehabilitation[tiab] OR "prehabilitation"[tiab])
```

D — Liver cirrhosis

```
("Liver Cirrhosis"[MeSH Terms] OR cirrhosis[tiab] OR
"Hepatic Encephalopathy"[MeSH Terms] OR "chronic liver disease"[tiab] OR
"hepatic sarcopenia"[tiab] OR "late evening snack"[tiab])
```

E — Cancer and cachexia

```
("Neoplasms"[MeSH Terms] OR cancer[tiab] OR "Cachexia"[MeSH Terms] OR
cachexia[tiab] OR gastrectomy[tiab] OR "perioperative"[tiab])
```

F — Chronic kidney disease and haemodialysis

```
("Renal Dialysis"[MeSH Terms] OR hemodialysis[tiab] OR haemodialysis[tiab] OR
"Renal Insufficiency, Chronic"[MeSH Terms] OR "chronic kidney disease"[tiab] OR
"protein-energy wasting"[tiab])
```

G — Neurological conditions

```
("Alzheimer Disease"[MeSH Terms] OR Alzheimer*[tiab] OR
"Cognitive Dysfunction"[MeSH Terms] OR "cognitive impairment"[tiab] OR
dementia[tiab] OR "Stroke"[MeSH Terms] OR stroke[tiab] OR
"Epilepsy"[MeSH Terms] OR epilep*[tiab] OR seizure*[tiab] OR
"Autism Spectrum Disorder"[MeSH Terms] OR autis*[tiab] OR
"traumatic brain injury"[tiab] OR kynurenine[tiab] OR "LAT1"[tiab])
```

H — Heart failure and orthopaedic conditions

```
("Heart Failure"[MeSH Terms] OR "heart failure"[tiab] OR
"Osteoarthritis, Knee"[MeSH Terms] OR osteoarthritis[tiab] OR
"Orthopedic Procedures"[MeSH Terms] OR orthopedic*[tiab] OR orthopaedic*[tiab])
```
